# Supplementary material for: The Toxoplasma micropore mediates endocytosis for selective nutrient salvage from host cell compartments
Source: Nat Commun. 2023 Feb 22;14:977. doi: 10.1038/s41467-023-36571-4 (PMC9947163; doi:10.1038/s41467-023-36571-4)
Supplement: Supplementary file 1 — Supplementary Information file [file 41467_2023_36571_MOESM1_ESM.pdf]

Supplementary Information

The *Toxoplasma* micropore mediates endocytosis for selective nutrient salvage from host cell compartments

Wenyan Wan[1, #], Hui Dong[1, #], De-Hua Lai[2, #], Jiong Yang[2], Kai He[1], Xiaoyan Tang[1], Qun Liu[1], Geoff Hide[3], Xing-Quan Zhu[4], L. David Sibley[5], Zhao-Rong Lun[2], and Shaojun Long[1, *]

[1]National Animal Protozoa Laboratory and School of Veterinary Medicine, China Agricultural University, Beijing 100193, China. LongS2018@163.com.

[2]State Key Laboratory of Biocontrol, School of Life Sciences, Sun Yat-Sen University, Guangzhou 510275, China.

[3]Biomedical Research and Innovation Centre and Environmental Research and Innovation Centre, School of Science, Engineering and Environment, University of Salford, Salford, M5 4WT, UK.

[4]College of Veterinary Medicine, Shanxi Agricultural University, Taigu 030801, Shanxi Province, China.

[5]Department of Molecular Microbiology, Washington University School of Medicine in Saint Louis, Saint Louis, MO 63110-1093, US.

[#]These authors contributed equally to the work

*Correspondence: LongS2018@163.com

This Supplementary Information includes

Supplementary Figures 1-12 and Legends;

Supplementary Table 1;

References.

Supplementary Figures 1-12 and Legends

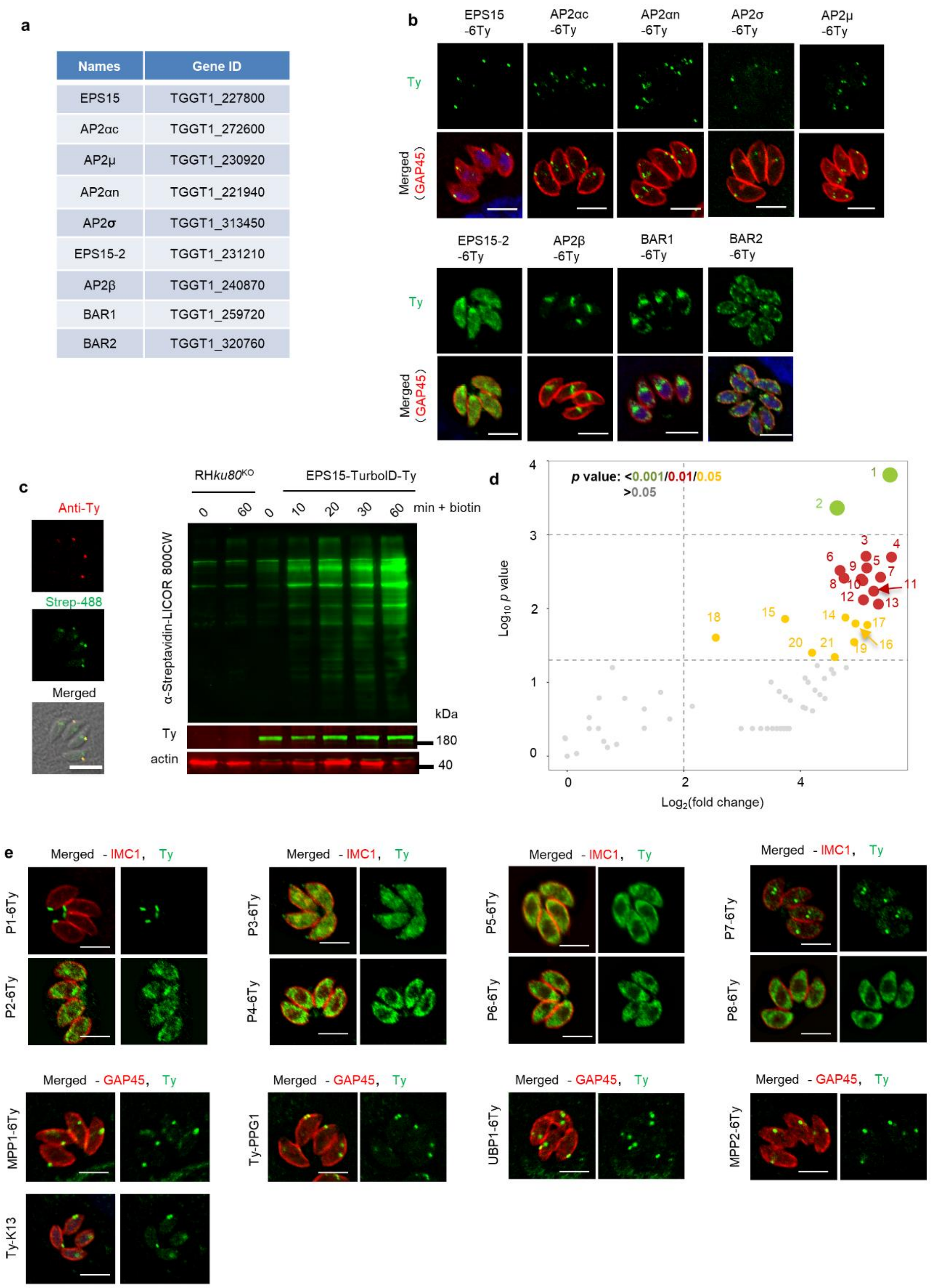

Supplementary Fig. 1. Discovery of proteins that are likely to be associated with endocytosis in *T. gondii*.

a Table list of proteins likely to be associated with endocytosis in *T. gondii*.

b Proteins listed in (**a**) were localized to discrete spots on the plasma membrane or to other structures, showing IFA results with Ty-tagged proteins (green) and GAP45 (red) as the control. Scale = 5 μm.

c IFA and western blot detection of TurboID labeling by the fusion of EPS15. Biotinylated proteins were detected using Streptavidin Alexa Fluor-488 (Strep-488) in the parental and TurboID fusion parasites grown for 24 hr and treated with 500 μM biotin for 1 hour. The Strep-488 signals (green) were co-localized with Ty staining (red) at the pellicular foci and additional green foci were the signals from the apicoplast. Western blot detection using streptavidin LICOR 800CW were performed for the parasites grown and treated with biotin for different times as indicated. ALD, aldolase as a control. Scale = 5 μm.

d Volcano plot analysis comparing the TurboID fusion to the control line RH (fold change > 2) using replicate mass-spectrometry datasets. Hits were analyzed by two-sided student t test, and significant hits were shown in numbers and indicated by colors. Detailed information on the hits were listed in Supplementary Table 4.

e IFA analysis of candidates identified in the biotinylation analysis. The candidates in (**d**) (Supplementary Table 4) were endogenously tagged with 6Ty for IFA analysis using Ty antibodies (green) and IMC1 (red), followed by secondary antibodies conjugated with Alexa Fluor reagents. Five new proteins were localized at the discrete spots similar to the bait EPS15, as observed in (**b**).

b, **c**, **e**, three independent experiments were performed for IFA and western blots with similar outcomes, and representative images are shown.

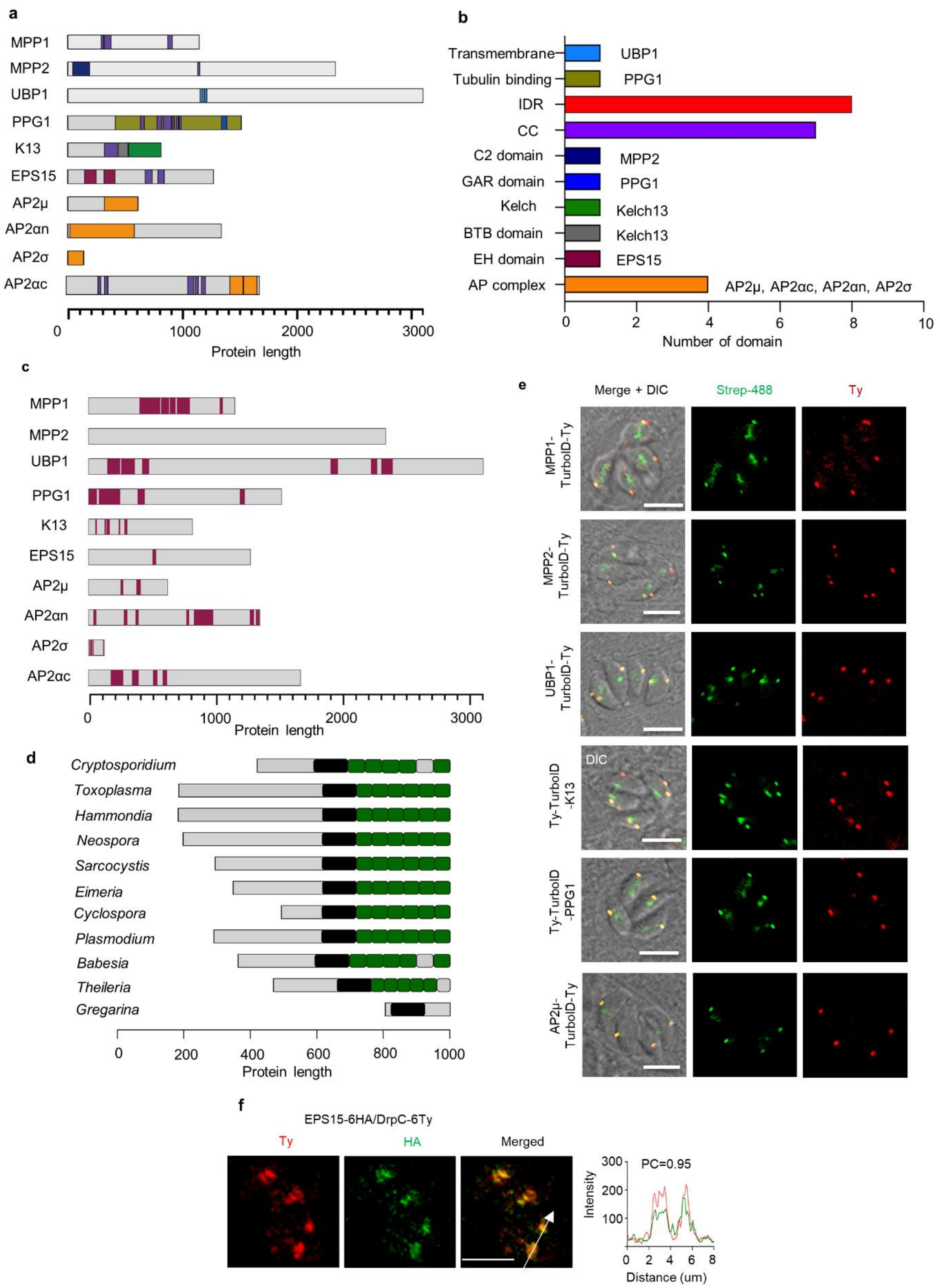

Supplementary Fig. 2. Domains of micropore proteins and the generation of TurboID fusion lines in *T. gondii*.

a-b Protein domain architectures were predicted by InterPro. Different domains were

mapped in the proteins (**a**) in colors. The domain names and numbers were shown in the same colors by columns (**b**). The domains of CC and IDR were universally present, but not mapped in the proteins. IDR, intrinsic disordered region; CC, coiled-coil domain.

c Intrinsic disordered regions were predicted by a newly developed webserver flDPnn[1]. This webserver predicts the intrinsic disordered regions together with the possible protein binding, DNA binding and RNA binding. Protein binding were found for proteins of K13, AP2αn, MPP1, UBP1, PPG1 and AP2αc.

d Domain architecture of Kelch13 in the representative species of apicomplexan. The species and accession numbers were the same to those in the Fig. 2a. Protein and domain sizes are scaled. The green box depicts the kelch domains while the black box indicates the BTB domain.

e Generation and verification of endogenous TurboID fusions, showing IFA staining for TurboID-Ty fusions (red), and for biotinylation test of TurboID detected by streptavidin Alexa Fluor-488 (Strep-488) in parasites. The biotinylated proteins (green) were co-localized with the Ty fusion (red), and the additional green spots were the apicoplast. The parasites were grown in D5 for 24 hours, and treated with 500 μM biotin for 1 hour before fixation for IFA. AP2μ-TurboID was used as a representative for the AP2 components, as other TurboID fusions were not generated successfully. GAP45 was stained green as a control for the IFA. Scale = 5 μm.

f Co-localization of DrpC with EPS15 by confocal microscopy. The endogenously tagged fusion line was generated and processed for IFA. Matured parasites were imaged, and Pearson Correlation (PC) for intensity of the arrow-headed line was analyzed. Scale = 5 μm.

e-f, Three independent experiments were performed with similar outcomes, and representatives of the images were shown.

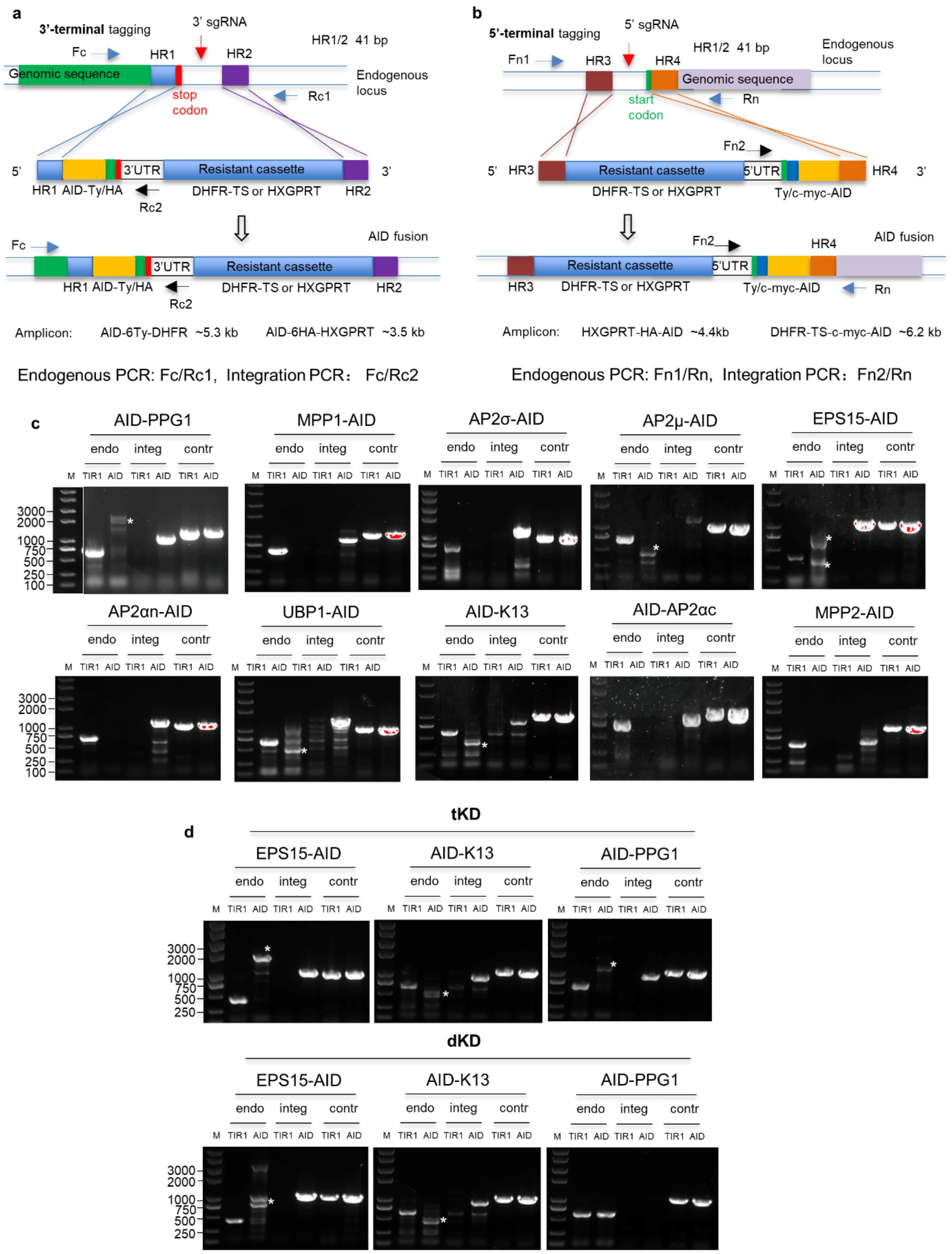

Supplementary Fig. 3. Confirmation of the AID lines generated by CRISPR-Cas9. **a-b** Illustration of strategy of strain generation by CRISPR-Cas9. The strategy of gene fusion by CRISPR-Cas9 exactly followed the approach developed previously in

another study[2]. The sgRNA either targeting to the upstream of start codon or the downstream of stop codon was expressed by a transient expression plasmid of Cas9. The Cas9-sgRNA system specifically creates a double strand break (DSB), which facilitates efficient integration of an amplicon containing either a AID-Ty/HA fragment for a 3'-terminal tagging at a specific gene or a Ty/c-myc-AID fragment for a 5'-terminal tagging at a specific gene, as illustrated in the figures. The integration of the fragment was guided by the homologies (HR1/2 for the 3'-terminal tagging and HR3/4 for the 5'-terminal tagging) from the specific gene. The diagnostic PCR was performed by primers from upstream and downstream of the homologies, and from the fragments, as illustrated. The genes encoding Kelch13, PPG1 and AP2αc were fused with the AID fragment at the 5'-termini. These fusions were termed as AID-Kelch13, AID-PPG1 and AID-AP2αc.

c-d Diagnostic PCR of the AID lines. The endogenous (endo) and integration (integ) PCRs were performed using primers illustrated in (**a-b**), using a general TAQ DNA polymerase with a PCR extension time of 1 minute. DNA used for the PCR was extracted from the TIR1 and AID lines, and the promoter region of tubulin was used as the PCR control (contr). Three independent experiments were performed with similar outcomes.

The endo PCR detected the specific endogenous DNA in the genome in the TIR1 line, but not in the AID lines, while the integ PCR detected the gene fusion in the AID lines but not in the TIR1 line. The diagnostic PCR were performed for three genes (i.e. EPS15, K13 and PPG1) in the lines of dKD and tKD. The stars (*) indicated the nonspecific bands for the endoPCR products. We observed that the endoPCR products for EPS15-AID (3' tagging) in the single AID (EPS15-AID), dKD and tKD appeared to be different. It has to be noted that the EPS15-AID line served as the parental line for the generation of the double AID (dKD) line and the triple AID (tKD) line. In addition, the nonspecific bands in the endoPCR for AID-PPG1 (5' tagging) in the single AID line (AID-PPG1) and the tKD line looked different as well. Compared to the PCR with the TIR1 samples that contain specific priming sequences, the endoPCR in the AID samples seemed to readily produce non-specific products and even differently produced non-specific products using different amounts of DNA in the sample. This is likely the reason for why we generally see more than one non-specific band. The diagnostic PCR clearly detected the specific PCR products (integ PCR)

from the AID integration in the AID parasites, but not in the parental line TIR1. We thus conclude that the AID lines were clean clones that contained a specific integration of AID at the targeted genes of interest used in this study.

In addition, the primers for the endoPCR could still amplify a long PCR product that contains the integrated heterologous DNA fragment (the AID-resistant cassettes with sizes > 4.0 kbp). However, the reaction was performed with a general TAQ DNA polymerase in a short extension time. This won't allow the PCR to make the long PCR products, but might produce different lengths of DNA products. This would thus produce complex fragments of DNA in different reactions.

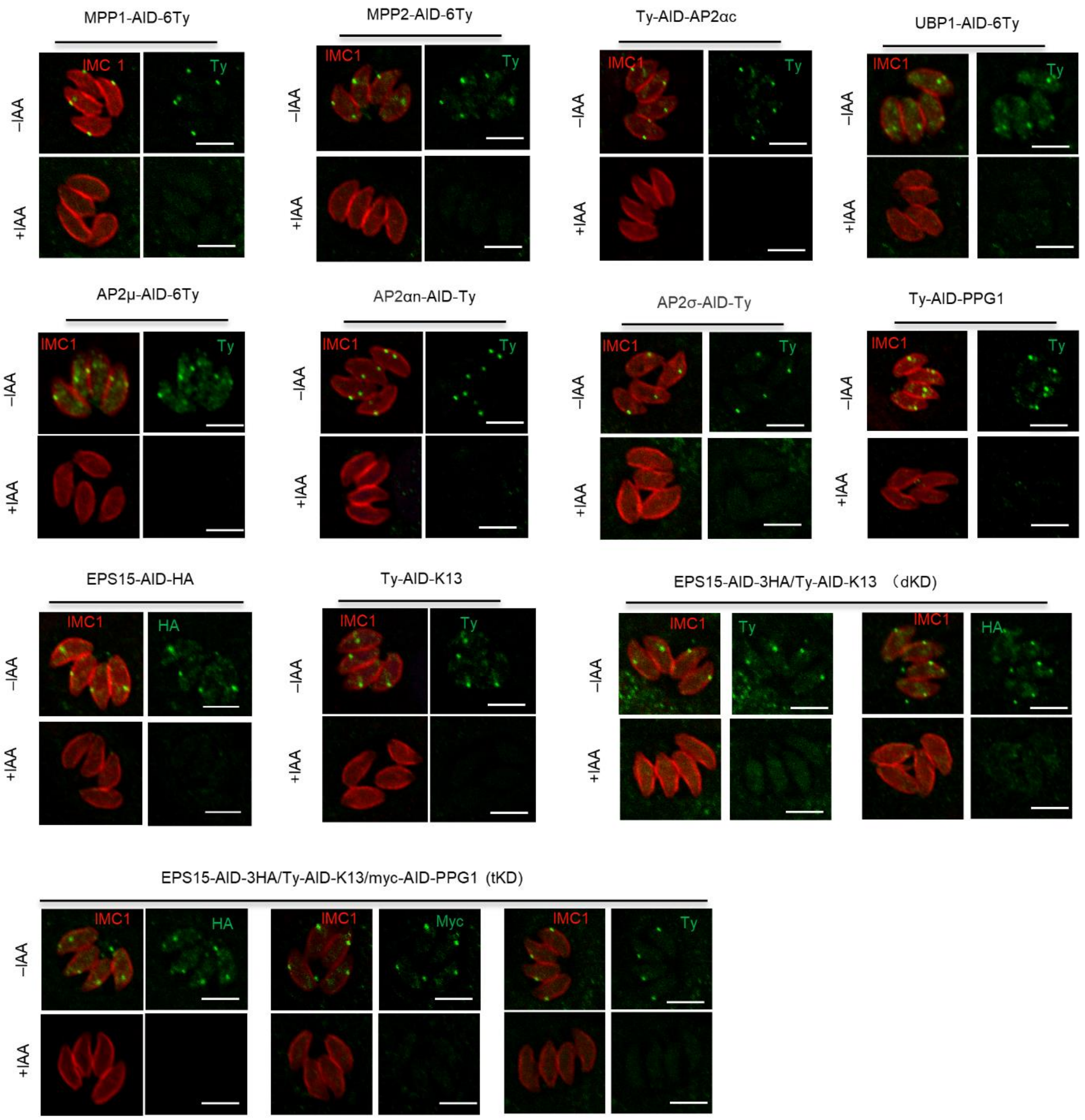

Supplementary Fig. 4. IFA verification of all the AID lines used in the study. Parasites were grown in ±IAA for 24 hr, followed by IFA analyses using antibodies against either Ty, HA or myc epitope fused with the AID degron. IMC1 served as a control for IFA. Scale = 5 μm. Three independent experiments were performed with similar outcomes, and one representative for each line was shown.

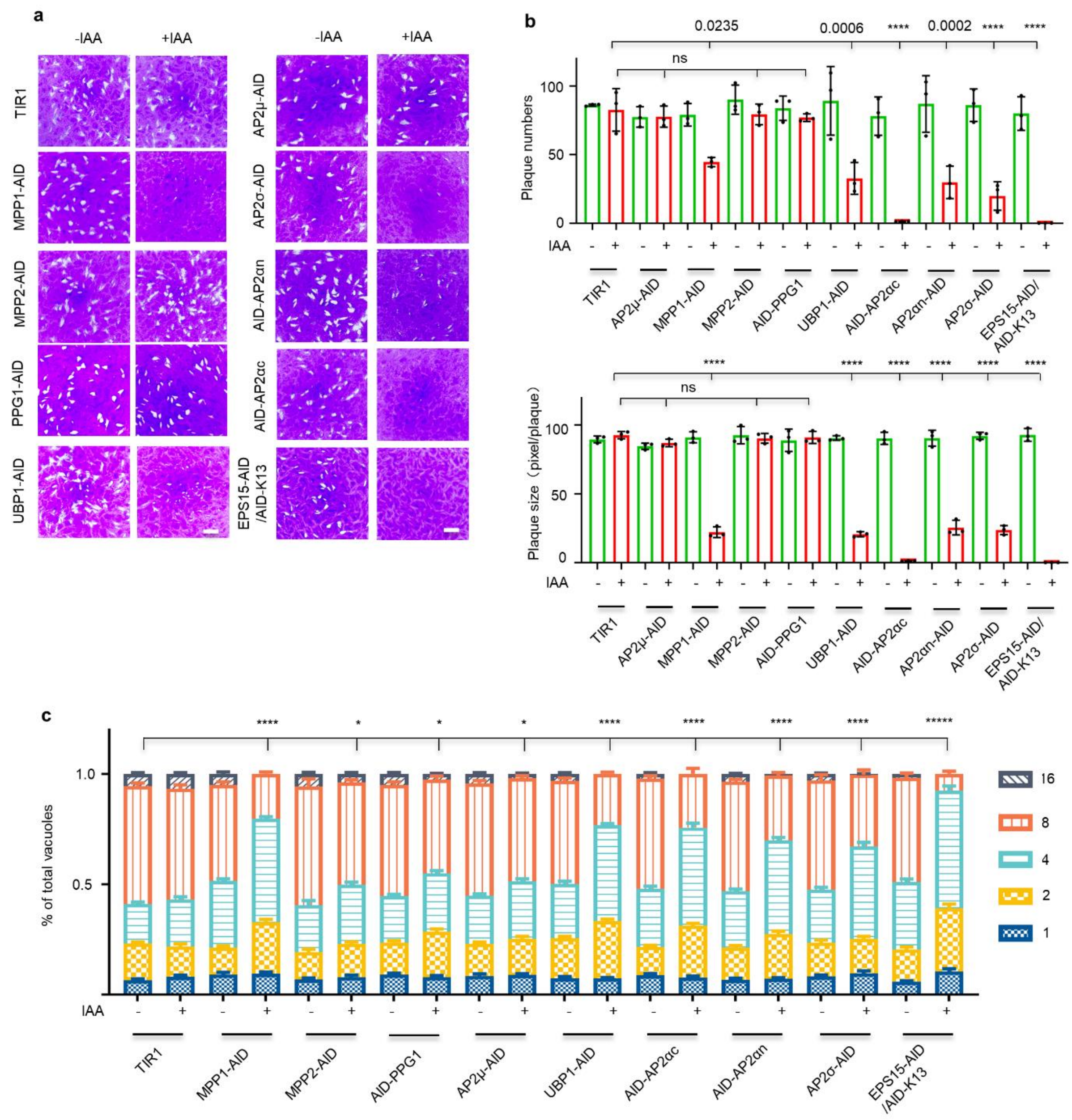

Supplementary Fig. 5. Plaque formation and replication of AID lines grown in ±IAA. **a**–**b** Parasite lines were grown on HFF monolayers in 6-well plates in ±IAA for 7 days, fixed and stained by crystal violet (**a**). One representative image from three independent experiments were shown for each line. Plaque numbers and sizes formed were measured and plotted (**b**). Three independent experiments with three replicates were performed with similar outcomes, and plaque sizes were measured for each independent experiment (n=18). Shown are the mean ± SD from three independent experiments and one-way ANOVA with Tukey's multiple comparison were performed, ***, *p*<0.0001, and other exact *p* values with significance were

provided. ns, not significant. Scale bar = 0.5 cm.

c Parasite replication for the lines grown in ±IAA for 24 hours. Vacuoles with different parasite numbers (1, 2, 4, 8 and 16) were counted (>100 vacuoles for each replicate), and three independent experiments were performed with triplicates. Data were plotted with mean ± SD of ratio of different numbers of parasites/vacuole in the total. The data were analyzed by two-way ANOVA with multiple comparison tests with adjustments. In comparing AID lines + IAA vs TIR1 +IAA, $p<0.0001$ for MPP1-AID, UBP1-AID, AID-AP2αc, AP2αn-AID, AP2σ-AID, EPS15/AID-K13 lines with ≥2 parasites/vacuole, and $p<0.05$ for MPP2-AID, AID-PPG1 and AP2μ-AID lines with 4-8 parasites/vacuole, while only the EPS15-AID/AID-K13 line with 1 and 2 parasite/vacuole was significant with $p<0.0001$.

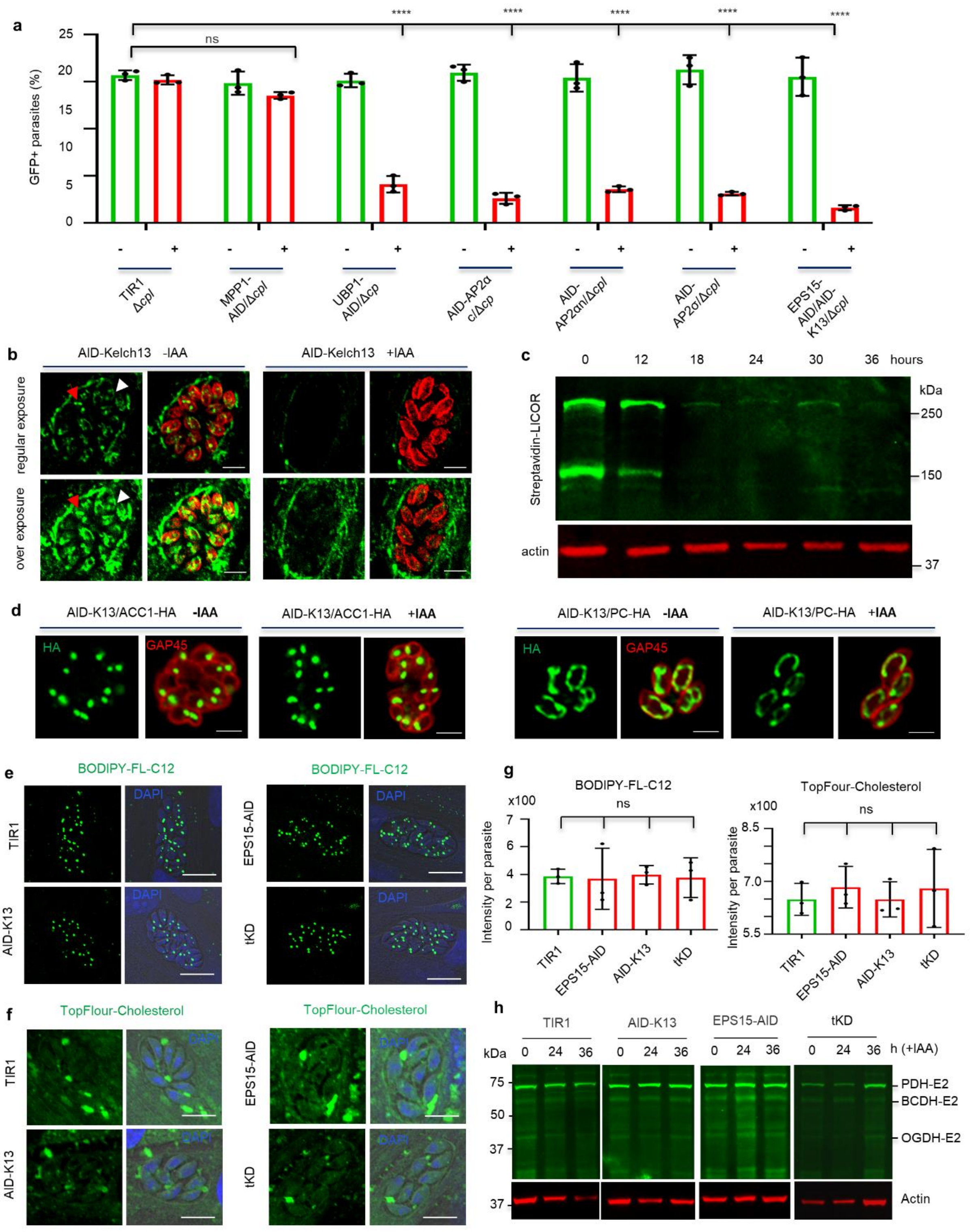

Supplementary Fig. 6. The micropore mediates parasite uptake of host cell cytosolic GFP, but not fatty acid and cholesterol.

a GFP uptake from host cell cytosol dramatically dropped in +IAA induced lines, showing percentage of GFP^{+} parasites in the population of the lines in IAA or the vehicle. Extracellular parasites mechanically lysed from parasites grown in GFP-

expressing host cells in ± IAA for 36 hours were adhered to poly-lysine-coated, permeabilized with 0.25% Triton X-100, and stained by DAPI for observation and counting. The parasite lines were edited to delete the *cpl* gene (Δ*cpl)* so that the parasites can accumulate host cell-derived GFP for observation. Data are shown with mean ± SD for three independent experiments with three replicates (N>100 parasites analyzed in each replicate), and analyzed with one-way ANOVA with Tukey's multiple comparison, ****, p<0.0001, ns, not significant.

b Detection of biotinylated proteins in the AID-K13 line. The line grown in auxin or the vehicle for 36 hours were processed with IFA using streptavidin-Alexa Flour-488 (green) and the control staining with GAP45 (red). The parasites were captured under regular and high exposure for both the induced and non-induced lines. The residue of ACC1 (with the strongest signal among three of the carboxylases) was observed only under high exposure in the induced parasites. The red and white arrows indicated the staining of ACC1 and PC, respectively. Scale = 5 μm.

c Western blots of biotinylated proteins in the AID-K13 line. The parasites were grown in HFF for 36 hours, and induced in auxin for 0, 12, 18, 24, 30 and 36 hours prior to harvesting for Western blots. The Western blots were performed using streptavidin-LICOR, to detect biotinylated proteins of ACC1 (the apicoplast carboxylase, 288 kDa) and PC (the mitochondrial carboxylase, 152 kDa). Actin served as the loading control.

d IFA detection of endogenously biotinylated proteins ACC1 and PC in the AID-K13 line grown in ± auxin for 36 hours. The proteins ACC1 and PC were tagged with 6HA via a CRISPR-Cas9 approach. Scale=5 μm.

e–**g** IFA detection of Bodipy FL-C12 and TopFluor-Cholesterol uptake by parasites grown in +IAA for 36 hours, showing representative images of the fluorescence after treatment of the parasites with Bodipy FL-C12-BSA (**e**), and TopfFluor-Cholesterol-LDL (parasites were grown in media without lipoproteins prior to pulsing with the complex) (**f**) for 30 min. The fluorescence in the vacuoles was evaluated, and all vacuoles containing 8 parasites on images were selected and measured

automatically by the system, and plotted as intensity per parasite. Three independent experiments were performed with triplicates, and ≥30 vacuoles were measured for each independent experiment. Data are shown as a mean ± SD with one-way ANOVA with Tukey's multiple comparison tests with adjustments. Scale bar = 10 μm, ns, not significant.

h Western blot detection of lipoic acid coupled with proteins in the parasites grown in ±IAA for 24 hours and 36 hours, showing a representative of the blots and actin serving as the control. Both mitochondrial and apicoplast lipolic acid-containing proteins were detected on the blots. The samples were derived from the same experiment and blots were processed in parallel. Three independent experiments were performed for each line.

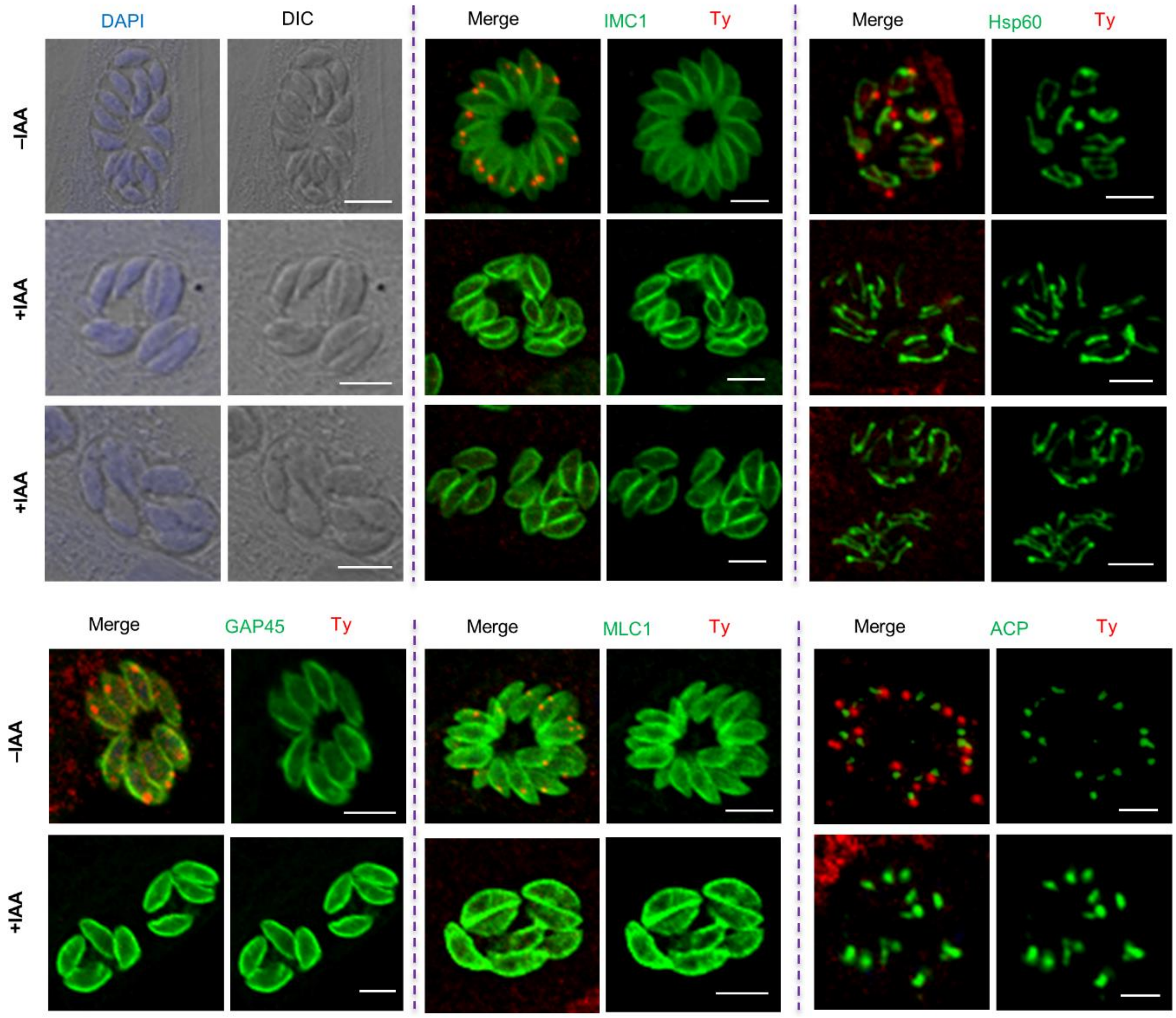

Supplementary Fig. 7. IFA analyses of cellular structures in K13-depleted parasites. Parasites were grown in the presence (+IAA) or absence (-IAA) of auxin for 36 hours, followed by IFA analyses using antibodies against IMC1, GAP45, MLC1, Hsp60 and ACP. IMC1, GAP45 and MLC1 serve as the markers for the inner membrane complex, while Hsp60 and ACP are the markers for the mitochondrion and the apicoplast, respectively. The Ty staining (red) in parasites was used for detection of Ty-AID-K13 in the parasites in the presence or absence of auxin. The rosette orientation of parasites appeared to be lost in majority of the parasitophorous vacuoles grown in auxin, as demonstrated by the DIC and the antibody staining. Three independent experiments were performed with similar outcomes, and representatives of the images were shown. DIC, differential interference contrast. Scale = 5 μM.

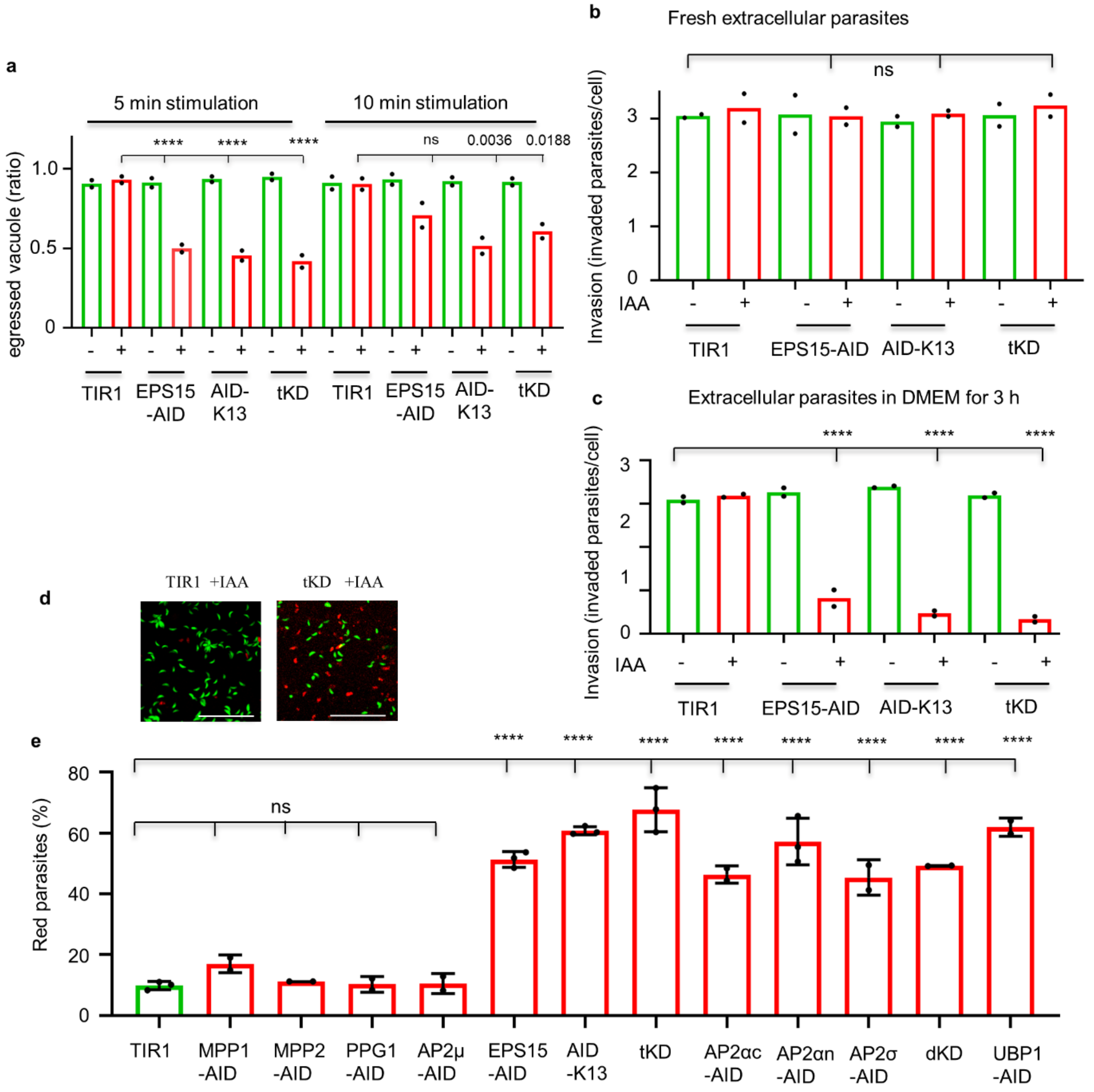

Supplementary Fig. 8. Parasites depleted with the micropore proteins were viable but lost fitness in the extracellular environment.

a Parasite egress was delayed in the lines grown in auxin for 36 hours. Parasites were stimulated by 3 μM A23187 for 5 min and 10 min, followed by IFA stains for GRA7 and IMC1 and by scorings of egressed and non-egressed vacuoles. The rates of egressed vacuoles at 10 min significantly increased compared to the rates at 5 min. Two independent experiments with triplicates were performed, and data are shown as a mean ± SD of the independent experiments with triplicates. At least 100 vacuoles were scored in each replicate.

b-c Extracellular parasites eventually lost fitness in protein-depleted parasites. Parasites were grown in HFF in ±IAA for 36 hours prior to mechanical egress for analysis of invasion capabilities. Freshly egressed parasites (**b**) and these parasites incubated in DMEM for 3 hours (**c**) were used to challenge HFF monolayers for 30 min at 37°C. The invaded and non-invaded parasites were scored (n>100 parasites in each replicate) and plotted as invaded parasites per host cell nuclei. Two independent experiments with triplicates were performed for the lines.
d-e Membrane integrity tests for extracellular parasites, showing example images with a green/red cell staining by probes from a kit (**d**). The intracellular parasites grown in auxin for 36 hours were mechanically egressed, followed by treatments with the dyes (live/dead cell imaging kit, ThermoFisher Scientific) on poly-lysine coated coverslips for 15 min. The stains were extended to all the AID lines for comparisons on integrity of parasite plasma membrane upon depletion of the corresponding proteins (**e**). Three independent experiments with triplicates were performed for TIR1, EPS15-AID, AID-K13 and tKD, while two independent experiments were performed for other AID lines.
Two-three independent experiments with triplicates (n>100 parasites in each replicate). Data are shown as a mean ± SD, and were analyzed by two-way ANOVA with multiple comparisons. ****, p<0.0001, and other exact p values with significance are provided; ns, not significant.

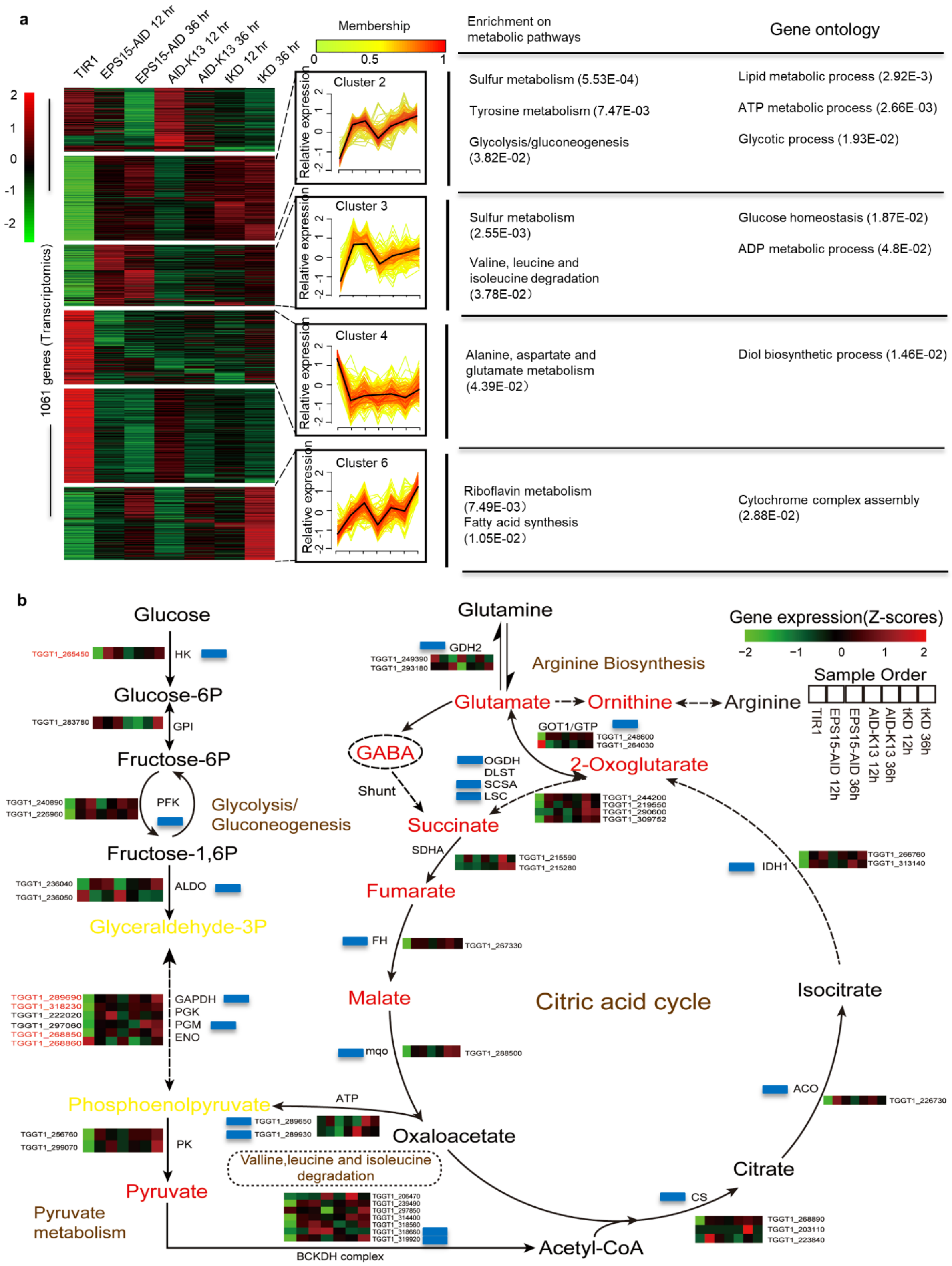

Supplementary Fig. 9. Parasite metabolic pathways were affected by depletion of micropore proteins.

a The Trend analysis of differential expression genes (DEGs) in +IAA induced lines

for 12 hours and 36 hours. To analyze overall changes, 6 of the experimental groups (EPS15-AID, AID-K13 and tKD lines induced at two time points) were compared the control TIR1 using the differential transcriptome (Supplementary Data 4). The fuzzy c-means algorithms was utilized for the analysis, which identified four characteristic Trend clusters. The membership of DEGs with different clusters was assessed in the analysis using the R-based tool. These clusters were used for an enrichment analysis in KEGG metabolic pathways and gene ontology (GO) functions, and representatives of the enrichments are shown in the figure, with details in Supplementary Table 10 and 11. The x axis represented seven samples from the lines, while the y axis represented log2-transformed, normalized intensity ratios in the lines. P values were listed for the enrichment of KEGG pathways and gene ontology (GO) from the TOXODB.

b Combined analyses of the -omics on the central carbon metabolism in *T. gondii*. The heatmap by the enzymes represented corresponding gene expression levels with the sample order at the right-upper corner. Gene expression was scaled using Z-scores for a mean value of three biological replicates. The metabolites shown in red were significant on the metabolomic analysis either in Experiment 1 or Experiment 2 with data analyzed by one-way ANOVA with Dunnett's multiple comparison, while the metabolites in yellow had *p* value >0.05 but close to that. The metabolites in black represent the ones not identified in the untargeted metabolomics, or not identified as differential metabolites. The gene ID in red represented significant changes on gene expression levels with data analyzed by DEseq package in R. The deep blue squares indicated that proteins were identified by mass-spectrometry on the TurboID fusion analysis (see Supplementary Data 7). Acetyl-CoA in the mitochondrion is produced by the catalysis of BCKDH complex with pyruvate or metabolites from valine, leucine and isoleucine[3], and the glycolysis and valine, leucine and isoleucine degradation were significantly enriched in the transcriptomic analysis.

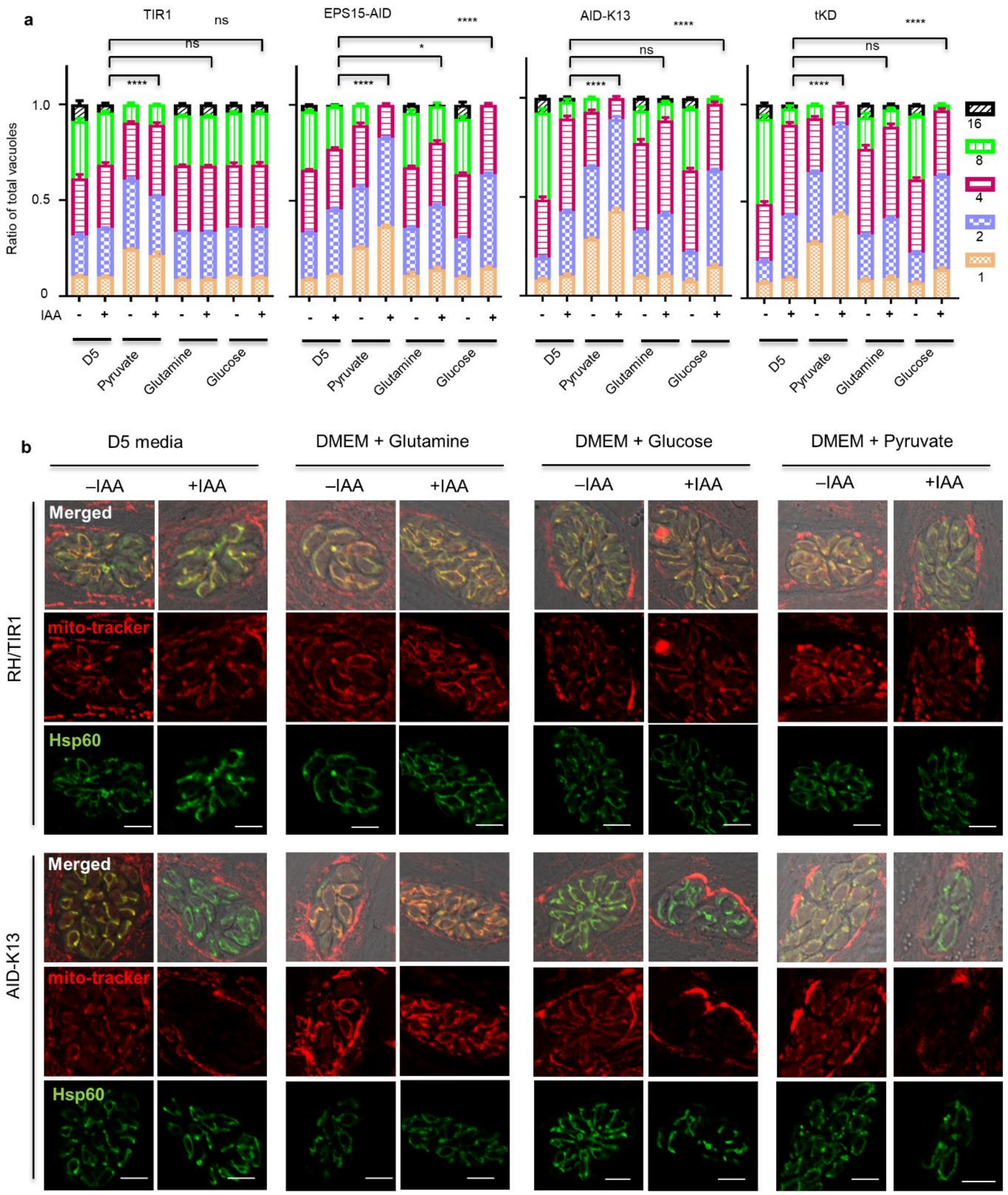

Supplementary Fig. 10. Confirmation of metabolic pathways by parasite replication assays and mitochondrial membrane potential in parasites grown in different carbon sources.

a The IAA induced AID lines grew less well in glucose than in glutamine. Parasites were grown in full media D5 or in media (A1443001, Gibco) containing only one carbon source with either 1.0 mM pyruvate, 2 mM glutamine or 25 mM glucose in the

presence of IAA or the vehicle for 30 hours, followed by IFA for recognition of parasites in vacuoles. The vacuoles containing different numbers of parasites (1, 2, 4, 8, 16) were counted, and expressed as a ratio of the vacuoles in the total. Data are shown as a mean ±SD for three experiments with three technical replicates (N>100 vacuoles for each replicate) and analyzed using two-way ANOVA with Tukey's multiple comparison test. In comparing the AID lines +IAA/glucose vs the corresponding AID lines +IAA/D5, $p<0.0001$ for 2 and 8 parasites/vacuole in all the AID lines, while not significant for 1-16 parasites/vacuole in the TIR1 +IAA/glucose vs TIR1 +IAA/D5; In comparing the AID lines +IAA/pyruvate vs the corresponding AID lines and TIR1 +IAA/D5, $p<0.0001$ for 1-8 parasites/vacuole. In comparing the AIDs +IAA/glutamine vs the AID lines +IAA/D5, $p<0.05$ for only 8 parasites/vacuole with EPS15-AID but not for other lines. Notably, the AID lines grown in media containing glucose did not have any vacuoles containing 16 parasites, which greatly differed to the same parasites grown in media containing glutamine.

b Mitochondrial membrane potential detected by mitotracker red was lost in AID-K13 when grown in D5 media and in media containing glucose, but recovered when grown in media containing glutamine, after induction in auxin for 30 hours. The intracellular parasites were grown in the media containing different carbon sources as described above. The parasites were then treated with 500 nM mitotracker red CMXRos (ThermoFisher, M7512) in DMEM for 30 min, followed by fixation, permeabilization and staining with rabbit antibodies against hsp60 and secondary antibodies conjugated with Alexa Fluor-488. Two independent experiments were performed with similar outcomes. Scale = 5 μm.

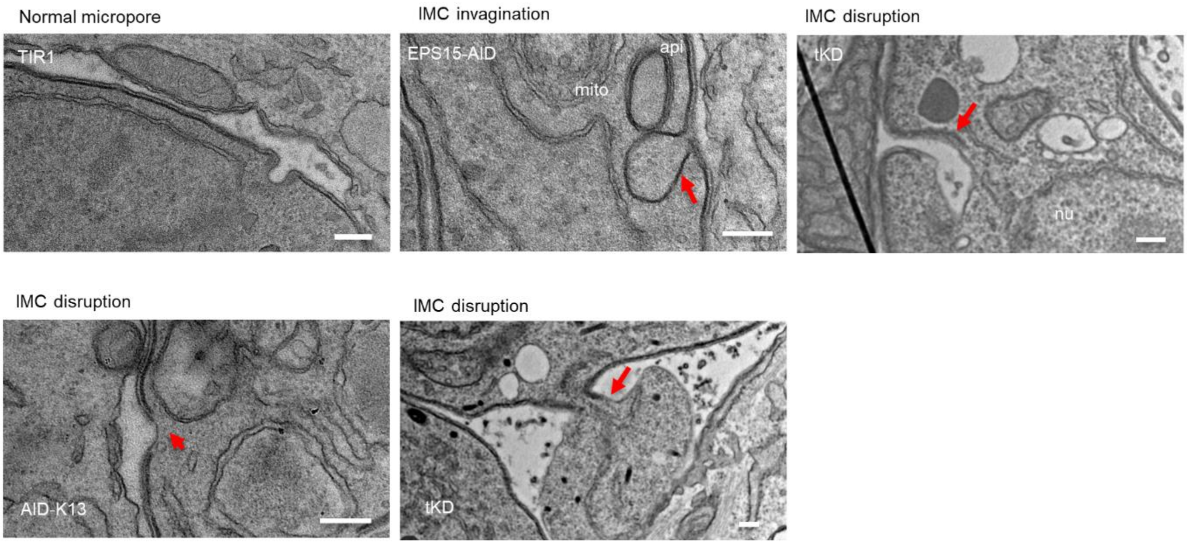

Supplementary Fig. 11. TEM analysis of serial sections for the key AID lines. Parasites were grown in HFF monolayers in IAA for 30 hours. The parasites fixed were serially sectioned for TEM. Representative of TEM images were shown for the normal micropore or disrupted structures (red arrowheads) from at least three independent experiments. The disrupted structures were judged by their locations in the parasites. The disrupted structures exhibited as follows: IMC invagination, and IMC disruption and the structure occasionally with cytosolic leaky. Scale bar = 200 nm. Go, Golgi; api, apicoplast; mito, mitochondrion, nu, nucleus.

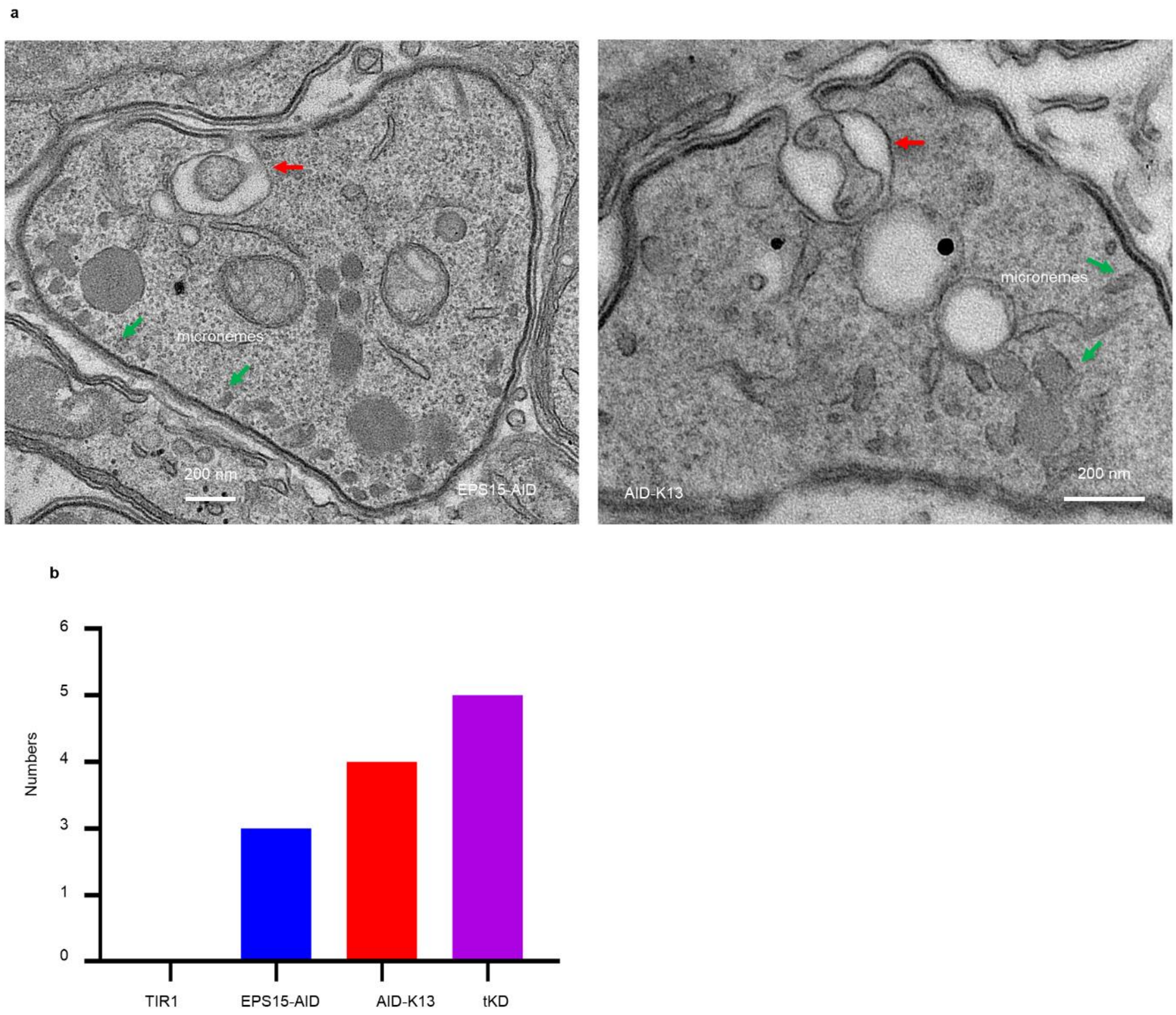

Supplementary Fig. 12. Observation of plasma membrane (PPM) invagination close to the apex in IAA induced lines.

a A PPM invagination (red arrowheads) close to the apex was observed in the IAA induced AID lines. This structure was previously reported in parasites treated with 200 μM oleic acid[4]. This PPM invagination in the IAA induced lines was characterized with its location close to the apex, as indicated by enriched presence of micronemes, and rhoptry neck (green arrowheads). The TEM clearly showed the invaginated plasma membrane without circling of electron dense area at the neck, however a stuck vesicle was observed inside the invagination. Scale = 200 nm.

b Quantification of the PPM invagination by conventional TEM. The PPM invagination close to the apex was quantified from the conventional TEM, which was performed for quantification of the micropore defects by checking 600 parasites. Similar

quantification outcomes were observed for the micropore defects and the PPM invagination close to the apex.

Supplementary Table 1. Comparison of proteins localized to the *T. gondii* micropore and *P. falciparum* cytostome.

Names (Tg)	Gene ID in Tg[①]	Essential for growth	GFP uptake [②]	Present in MS[③]	Names (Pf)[④]	Gene ID in Pf[⑤]	endocytosis in Pf[⑥]	Essential growth[⑥]
Kelch13	262150	Yes	Yes	Yes	Kelch13	1343700	Yes (ring)	Yes
EPS15	227800	partial	Yes	Yes	Eps15	1025000	Yes (troph)	Yes
UBP1	277895	partial	Yes	Yes	UBP1	0104300	Yes (troph)	Yes
AP2αc	272600	Yes (HFF)	Yes	Yes	KIC4	1246300	nd	No
PPG1	297520	No	nd	Yes	KIC2	1227700	nd	No
AP2μ	230920	No	nd	Yes	AP2μ	1218300	Yes (troph)	Yes
DrpC	270690	Yes [5]	nd	Yes	dynamin	1218500	unknown	unknown
AP2σ	313450	partial	Yes	Yes	AP2σ	0217300	unknown	unknown
AP2αn	221940	partial	Yes	Yes	AP2α	0617100	unknown	unknown
MPP1	202220	partial	No	Yes	n.a	n.a	n.a	n.a
MPP2	286030	No	nd	Yes	n.a	n.a	n.a	n.a
	265420	nd	nd	No	KIC3	0914400	nd	No
	257070	nd	nd	Yes	KIC7	0813000	Yes	Yes
	277560	nd	nd	No	KIC10	1122500	nd	No
	206490	nd	nd	No	MCA2	1438400	nd	No
	n.a	n.a	n.a	n.a	KIC1	0606000	nd	No
	n.a	n.a	n.a	n.a	KIC5	1138700	nd	No
	n.a	n.a	n.a	n.a	KIC6	0609700	nd	No
	n.a	n.a	n.a	n.a	KIC8	1014900	nd	No
	n.a	n.a	n.a	n.a	KIC9	1442400	nd	No

[①]refers to gene accession numbers for TgME49: http://ToxoDB.org
[②]refers to the GFP acquisition from the host cell HFF expressing GFP in this study.
[③]refers to the micropore proteomic datasets identified by the biotinylation approach.
[④]refers to the proteins reported in ref[6], or the names (dynamin, AP2σ and AP2α) from PlasmoDB: https://plasmodb.org/plasmo/app/
[⑤]refers to gene accession numbers for *P. falciparum* 3D7: https://plasmodb.org/plasmo/app/
[⑥]refers to the information reported in ref[6].
Orthologues were identified by searching the proteins in ToxoDB or PasmoDB and the hits with E-values <1e-7 were considered as orthologues. Note: nd, not determined; n.a, not applicable; Tg, *T. gondii*; Pf, *P.falciparum*.

References:

1. Hu, G. *et al.* flDPnn: Accurate intrinsic disorder prediction with putative propensities of disorder functions. *Nat Commun* **12**, 4438 (2021).
2. Long, S. *et al.* Calmodulin-like proteins localized to the conoid regulate motility and cell invasion by *Toxoplasma gondii*. *PLoS Pathog* **13**, e1006379 (2017).
3. Oppenheim, R.D. *et al.* BCKDH: the missing link in apicomplexan

mitochondrial metabolism is required for full virulence of *Toxoplasma gondii* and *Plasmodium berghei*. *PLoS Pathog* **10**, e1004263 (2014).
4. Nolan, S.J., Romano, J.D. & Coppens, I. Host lipid droplets: An important source of lipids salvaged by the intracellular parasite *Toxoplasma gondii*. *PLoS Pathog* **13**, e1006362 (2017).
5. Heredero-Bermejo, I. *et al.* TgDrpC, an atypical dynamin-related protein in *Toxoplasma gondii*, is associated with vesicular transport factors and parasite division. *Mol Microbiol* **111**, 46-64 (2019).
6. Birnbaum, J. *et al.* A Kelch13-defined endocytosis pathway mediates artemisinin resistance in malaria parasites. *Science* **367**, 51-59 (2020).
