# Supplementary material for: The Toxoplasma micropore mediates endocytosis for selective nutrient salvage from host cell compartments
Source: Nat Commun. 2023 Feb 22;14:977. doi: 10.1038/s41467-023-36571-4 (PMC9947163; doi:10.1038/s41467-023-36571-4)
Supplement: Supplementary file 3 — Description of additional Supplementary files [file 41467_2023_36571_MOESM3_ESM.docx]

**Description of Supplementary Data and Movies**

**Supplementary Movie 1**. Tomography of the *Toxoplasma* micropore that is budding an endocytic vesicle at its base*.* The tomography is reconstructed from a 260 nm thick section, from which the micropore section consists of 166 individual images in the movie. The 3D structure was modeled with colors on the components. Deep green indicates the parasite plasma membrane, deep red denotes the IMC, and yellow shows the proteins coated on the plasma membrane of the micropore.

**Supplementary Movie 2**. Filtered protein interactome generated by SFINX using TurboID mass spectrometry datasets. The datasets were statistically analyzed by hypergeometric test. The strictness was set at 4. Baits (TurboID fusion lines), deep blue nodes; Preys (interactors identified), light blue nodes.

**Supplementary Data 1**. Lines used in this study.

**Supplementary Data 2**. Plasmids used in this study.

**Supplementary Data 3**. Primers used in this study.

**Supplementary Data 4**. Mass-spectrometry datasets of EPS15-TurboID and the parental line.

**Supplementary Data 5.** Analysis of hits on the mass-spectrometry results with EPS15-TurboID and the parental line. Two-sided student t test was performed for the datasets.

**Supplementary Data 6**. Conservation of micropore proteins across the Apicomplexa.

**Supplementary Data 7**. Peptides related to the IMC and microtubules on the MS datasets of all TurboID fusions.

**Supplementary Data 8**. Basic data for the SFINX protein interaction analysis.

**Supplementary Data 9**. Summary of SFINX output with scores and *p* values. The core interactome was mediated by Kelch13, EPS15 and PPG1. The datasets were statistically analyzed by hypergeometric test.

**Supplementary Data 10**. Full transcriptomic datasets for the TIR1, AID-K13 and tKD lines induced by auxin.

**Supplementary Data 11**. The transcriptomic datasets of differential transcripts for the TIR1, AID-K13 and tKD lines induced by auxin.

**Supplementary Data 12**. KEGG enrichment for characteristic modules of the WGCNA co-expression networks analysis on the full transcriptomic datasets using the AID and parental lines grown in auxin. Statistical analysis was performed based on hypergeometric test.

**Supplementary Data 13**. KEGG enrichment for characteristic clusters of the Trend analysis on differential transcripts using the AID and parental lines grown in auxin. Statistical analysis was performed based on hypergeometric test.

**Supplementary Data 14.** GO enrichment for characteristic clusters of the Trend analysis on differential transcripts using the AID and parental lines grown in auxin. Statistical analysis was performed based on hypergeometric test.

**Supplementary Data 15**. Raw datasets of untargeted metabolomics in experiment 1.

**Supplementary Data 16**. Raw datasets of untargeted metabolomics in experiment 2.

**Supplementary Data 17**. Analysis of the differential metabolites in experiment 1 (n=5). The datasets in experiment 1 were analyzed with One-way ANOVA with Dunnett's multiple comparison test.

**Supplementary Data 18**. Analysis of the differential metabolites in experiment 2 (n=3). The datasets in experiment 2 were analyzed with One-way ANOVA with Dunnett's multiple comparison test.

**Supplementary Data 19**. MetaboAnalyst pathway analysis of the differential metabolites from untargeted metabolomics with the TIR1 and AID lines grown in auxin. Enriched pathways are listed for Experiment 1 and Experiment 2. Statistical analysis was performed based on hypergeometric test.

**Supplementary Data 20**. The datasets of biotinylated proteins by TurboID fusions with the micropore proteins. The original mass-spectrometry datasets were exported using the following settings: 0 peptides in the parental line, and ≥2 peptides in either of the TurboID fusions and peptide threshold = 95%.

**Supplementary Data 21.** Mass-spectrometry datasets of the proximity labeling analysis and the KEGG enrichment analysis. The datasets were exported with the following settings: 0 peptides from the parental line, and ≥2 peptides from either of the TurboID fusions (n=2 for each) and peptide threshold=95%. The datasets were used for an enrichment analysis using ClusterProfiler in R with KEGG of *T. gondii*. Statistical analysis was performed based on hypergeometric test.
