# Supplementary material for: The Toxoplasma micropore mediates endocytosis for selective nutrient salvage from host cell compartments
Source: Nat Commun. 2023 Feb 22;14:977. doi: 10.1038/s41467-023-36571-4 (PMC9947163; doi:10.1038/s41467-023-36571-4)
Supplement: Supplementary file 27 — Reporting Summary [file 41467_2023_36571_MOESM27_ESM.pdf]

nature portfolio

Corresponding author(s): Shaojun Long

Last updated by author(s): Jan 17, 2023

Reporting Summary

Nature Portfolio wishes to improve the reproducibility of the work that we publish. This form provides structure for consistency and transparency in reporting. For further information on Nature Portfolio policies, see our Editorial Policies and the Editorial Policy Checklist.

Statistics

For all statistical analyses, confirm that the following items are present in the figure legend, table legend, main text, or Methods section.

n/a	Confirmed	
☐	☒	The exact sample size (*n*) for each experimental group/condition, given as a discrete number and unit of measurement
☐	☒	A statement on whether measurements were taken from distinct samples or whether the same sample was measured repeatedly
☐	☒	The statistical test(s) used AND whether they are one- or two-sided<br>*Only common tests should be described solely by name; describe more complex techniques in the Methods section.*
☒	☐	A description of all covariates tested
☒	☐	A description of any assumptions or corrections, such as tests of normality and adjustment for multiple comparisons
☐	☒	A full description of the statistical parameters including central tendency (e.g. means) or other basic estimates (e.g. regression coefficient) AND variation (e.g. standard deviation) or associated estimates of uncertainty (e.g. confidence intervals)
☐	☒	For null hypothesis testing, the test statistic (e.g. *F*, *t*, *r*) with confidence intervals, effect sizes, degrees of freedom and *P* value noted<br>*Give P values as exact values whenever suitable.*
☒	☐	For Bayesian analysis, information on the choice of priors and Markov chain Monte Carlo settings
☒	☐	For hierarchical and complex designs, identification of the appropriate level for tests and full reporting of outcomes
☒	☐	Estimates of effect sizes (e.g. Cohen's *d*, Pearson's *r*), indicating how they were calculated

Our web collection on statistics for biologists contains articles on many of the points above.

Software and code

Policy information about availability of computer code

Data collection	Metabolomic data: Agilent 7890A/5975C GC-MS system (Agilent Technologies) (analyzed by Shanghai Proleader Biotech Co.)<br>Proteomic data: Mascot (Matrix Science, London, UK; version 2.6.2) (analyzed by NEBRASKA CENTER FOR BIOTECHNOLOGY, Nebraska, US)<br>Transcriptomic data: Illumina HiSeq2500 (analyzed by GENE DENOVO Biotechnology Co. Guangzhou, China)<br>Microscopy: NIKON Confocal C2+ and NIS Elements AR (NIKON)<br>DNA and protein gels: BIO-RAD ChemiDoc MP Imaging System
Data analysis	Image J version 1.52p, Scaffold_4.10.0 (Proteome Software Inc.), Omicsmart (GENE DENOVO BIOTECH), Prism version 8.0.2 (Graphpad Software Inc.), ChemiDoc MP imaging system (Bio-Rad), SnapGene 4.1.9, HISAT2 (v2.2.4), StringTie (v1.3.1), DEseq2 package in R (v1.24.0), Mfuzz Package in R (v2.48.0), WGCNA Package in R (v1.69), MetaboAnalyst 5.0 server, Bowties2 (v2.2.8), Fastp v0.18.0, SFINX Package in R. NIS Elements AR 5.11.01 (NIKON).

For manuscripts utilizing custom algorithms or software that are central to the research but not yet described in published literature, software must be made available to editors and reviewers. We strongly encourage code deposition in a community repository (e.g. GitHub). See the Nature Portfolio guidelines for submitting code & software for further information.

Data

Policy information about availability of data

All manuscripts must include a data availability statement. This statement should provide the following information, where applicable:

- Accession codes, unique identifiers, or web links for publicly available datasets
- A description of any restrictions on data availability
- For clinical datasets or third party data, please ensure that the statement adheres to our policy

Data availability
The proteomic and metabolomic data reported in this paper have been deposited in the OMIX, China National Center for Bioinformation/Beijing Institute of Genomics, Chinese Academy of Sciences (https://ngdc.cncb.ac.cn/omix) with accession numbers: OMIX001158 and OMIX001386, respectively. Minimally processed data of the proteomics and metabolomics data are available in Supplementary Data 1 and Data 2, and Supplementary Data 5 and Supplementary Data 6, respectively. Transcriptomics sequencing data are available in the short read archive (SRA) of database of National Center for Biotechnology Information under the accession number of PRJNA812267. Minimally processed data are available in Supplementary Data 3 and Data 4. Toxoplasma gondii genome information can be found in ToxoDB release 53 (http://toxodb.org) and Eukaryotic pathogen, Vector & Host Information Resources can be found in VEupathDB (http://veupathdb.org). Source data are provided in this paper with an accession link: https://github.com/He-Kai-fly/microspore_sourcedata.

Human research participants

Policy information about studies involving human research participants and Sex and Gender in Research.

Reporting on sex and gender	not applicable
Population characteristics	not applicable
Recruitment	not applicable
Ethics oversight	not applicable

Note that full information on the approval of the study protocol must also be provided in the manuscript.

Field-specific reporting

Please select the one below that is the best fit for your research. If you are not sure, read the appropriate sections before making your selection.

[x] Life sciences [ ] Behavioural & social sciences [ ] Ecological, evolutionary & environmental sciences

For a reference copy of the document with all sections, see nature.com/documents/nr-reporting-summary-flat.pdf

Life sciences study design

All studies must disclose on these points even when the disclosure is negative.

Sample size

Sample sizes were not predetermined, but were chosen to reflect biological and technical variance of the investigated parameters based on previously published literature (1,2, 3). Experiments were generally repeated at least three times. When additional experiments were performed, all experiments are reported. Sample sizes are consistent with similarly published studies (1, 2, 3).

1. Brown, K. M. & Sibley, L. D. Essential cGMP Signaling in Toxoplasma Is Initiated by a Hybrid P-Type ATPase-Guanylate Cyclase. Cell Host Microbe 24, 804-816 e806, doi:10.1016/j.chom.2018.10.015 (2018).
2. Long, S., Anthony, B., Drewry, L.L. & Sibley, L.D. A conserved ankyrin repeat-containing protein regulates conoid stability, motility and cell invasion in Toxoplasma gondii. Nature Communications. 8, 2236 (2017).
3. Waldman BS, Schwarz D, Wadsworth MH 2nd, Saeij JP, Shalek AK, Lourido S. Identification of a Master Regulator of Differentiation in Toxoplasma. Cell. 23;180(2):359-372.e16 (2016).

Data exclusions

No exclusion criteria were predetermined and no data were excluded.

Replication

All experiments were performed in at least three biological replicates to allow relevant statistical analysis and several technical replicates were generated to ensure data reproducibility. All microscopy images are representatives of at least three independent experiments and all experiments resulted in comparable results.

Randomization

Assignment of strains to treatment groups was randomized between biological replicates. Mice were randomly assigned to experimental

	groups
Blinding	Investigators were blinded during data acquisition and analysis. The analyses performed have quantitative endpoints and are not subject to investigator bias.

Reporting for specific materials, systems and methods

We require information from authors about some types of materials, experimental systems and methods used in many studies. Here, indicate whether each material, system or method listed is relevant to your study. If you are not sure if a list item applies to your research, read the appropriate section before selecting a response.

Materials & experimental systems

n/a	Involved in the study	
☐	☒	Antibodies
☐	☒	Eukaryotic cell lines
☒	☐	Palaeontology and archaeology
☐	☒	Animals and other organisms
☒	☐	Clinical data
☒	☐	Dual use research of concern

Methods

n/a	Involved in the study	
☒	☐	ChIP-seq
☒	☐	Flow cytometry
☒	☐	MRI-based neuroimaging

Antibodies

Antibodies used

Rabbit polyclonal anti-HA (ThermoFisher, 71-5500, the polyclonal antibodies against
A synthetic peptide corresponding to the nine amino acid sequence HA-tag YPYDVPDYA), 1:500; Mouse monoclonal anti-HA (BioLegend, anti-HA, clone 16B12, 901501, the monoclonal antibodies were raised against Monoclonal antibody HA.11 (HA, 16B12, flu tag) was raised against the twelve amino acid peptide CYPYDVPDYASL), 1:500; Mouse monoclonal anti-c-myc (9E10)(MA1-980, ThermoFisher Scientific, the monoclonal antibodies were raised against Synthetic peptide A(408) EEQKLISEEDLLRKRREQLKHKLEQLRNSCA(438) of human c-Myc), 1:500; Mouse monoclonal anti-Ty1 (clone BB2)(Bastin et al., 1996), 1:500; Rabbit anti-lipoic acid antibodies, ab58724 from Abcam; 1:250. Anti-Ty1, BB2, mouse monoclonal antibodies, dilution: 1:500.

Rabbit anti-GRA7 antibodies (1:500); Rabbit or mouse polyclonal anti-GAP45, ACP, IMC1, hsp60 and actin were generated in our lab, all were used at 1:500.

Goat anti-Rabbit or Mouse Secondary antibodies conjugated with Alexa Fluors, ThermoFisher Scientific, A-11029, A-11031, A-11034, A-11036, 1:1000; goat anti-mouse IgG (H+L) Alexa Flour-647 antibody (A21235, ThermoFisher Scientific), 1:1000; Goat Anti-Rabbit IgG F(ab')2 fragment - Atto 488 (Sigma-Aldrich, 36098), 1:1000;

IRDye 680CW or 800CW anti-Mouse or anti-Rabbit (LICOR, 926-32210, 926-32211, 926-68070, 926-68071, #926-32230), 1:1000; streptavidin Alexa Fluor-488 (926-3230, LICOR), 1:500; streptavidin LICOR CW800 (926-32230, LICOR), 1:500.

Alexa FluorTM 488 goat anti-mouse IgG, 10 nm colloidal gold (Invitrogen, A31561), 1:1000

Validation

Mouse or Rabbit anti-HA, anti-c-myc were generally used in studies of Toxoplasma gondii and other species. Brown, K. M. & Sibley, L. D. Essential cGMP Signaling in Toxoplasma Is Initiated by a Hybrid P-Type ATPase-Guanylate Cyclase. Cell Host Microbe 24, 804-816 e806, doi:10.1016/j.chom.2018.10.015 (2018).Brown, K.M., Long, S. & Sibley, L.D. Plasma Membrane Association by N-Acylation Governs PKG Function in Toxoplasma gondii. mBio 8(2017); Long, S., Brown, K.M., Drewry, L.L., Anthony, B., Phan, I.Q.H., and Sibley, L.D. (2017). Calmodulin-like proteins localized to the conoid regulate motility and cell invasion by Toxoplasma gondii. PLoS Pathog 13, e1006379; Long, S., Anthony, B., Drewry, L.L. & Sibley, L.D. A conserved ankyrin repeat-containing protein regulates conoid stability, motility and cell invasion in Toxoplasma gondii. Nature Communications. 8, 2236 (2017). The correct Western blot bands and IFA localizations were seen in all the lines that contain fusion proteins with the eptitope tags in previous studies and in this study.

Rabbit anti-GRA7 antibodies was generated in a previous study. Alaganan, A., Fentress, S.J., Tang, K., Wang, Q. & Sibley, L.D. Toxoplasma GRA7 effector increases turnover of immunity-related GTPases and contributes to acute virulence in the mouse. Proc. Natl. Acad. Sci. U. S. A. 111, 1126-31 (2014).

Mouse monoclonal BB2 anti-Ty was generated in a previous study. Bastin P, Bagherzadeh Z, Matthews KR, Gull K. A novel epitope tag system to study protein targeting and organelle biogenesis in Trypanosoma brucei. Mol Biochem Parasitol. 77(2):235-9. doi: 10.1016/0166-6851(96)02598-4.

Streptavidin reagents for IFA and Western blot were used in previous studies with biotin labeling. Nadipuram SM, Kim EW, Vashisht AA, Lin AH, Bell HN, Coppens I, Wohlschlegel JA, Bradley PJ. In Vivo Biotinylation of the Toxoplasma Parasitophorous Vacuole Reveals Novel Dense Granule Proteins Important for Parasite Growth and Pathogenesis. mBio. 2016 Aug 2;7(4):e00808-16. doi: 10.1128/mBio.00808-16. Long, S., Brown, K.M., Drewry, L.L., Anthony, B., Phan, I.Q.H., and Sibley, L.D. (2017). Calmodulin-like proteins localized to the conoid regulate motility and cell invasion by Toxoplasma gondii. PLoS Pathog 13, e1006379;

For antibodies (IMC1, actin, ACP, hsp60 and GAP45) newly generated in our own lab, Western blots bands were seen at the correct molecular weights on Western blots, and correct cellular localizations were seen on IFA analyses. For the information of these

proteins, see the literatures belows:
For GAP45 information: Frenal, K. et al. Functional dissection of the apicomplexan glideosome molecular architecture. Cell Host Microbe 8, 343-357, doi:10.1016/j.chom.2010.09.002 (2010).
For IMC1 information: Wichroski, M. J., Melton, J. A., Donahue, C. G., Tweten, R. K. & Ward, G. E. Clostridium septicum alpha-toxin is active against the parasitic protozoan Toxoplasma gondii and targets members of the SAG family of glycosylphosphatidylinositol-anchored surface proteins. Infect Immun 70, 4353-4361, doi:10.1128/IAI.70.8.4353-4361.2002 (2002).
For Actin information: Periz, J. et al. A highly dynamic F-actin network regulates transport and recycling of micronemes in Toxoplasma gondii vacuoles. Nat Commun 10, 4183, doi:10.1038/s41467-019-12136-2 (2019).
For ACP information: Mazumdar, J., E, H. W., Masek, K., C, A. H. & Striepen, B. Apicoplast fatty acid synthesis is essential for organelle biogenesis and parasite survival in Toxoplasma gondii. Proc Natl Acad Sci U S A 103, 13192-13197, doi:10.1073/pnas.0603391103 (2006).
For hsp60 information: Toursel, C., Dzierszinski, F., Bernigaud, A., Mortuaire, M. & Tomavo, S. Molecular cloning, organellar targeting and developmental expression of mitochondrial chaperone HSP60 in Toxoplasma gondii. Mol Biochem Parasitol 111, 319-332, doi:10.1016/s0166-6851(00)00324-8 (2000).

Eukaryotic cell lines

Policy information about cell lines and Sex and Gender in Research

Cell line source(s)	HFF-1 (ATCC, SCRC-1041): Sidik SM, Huet D, Ganesan SM, Huynh MH, Wang T, Nasamu AS, Thiru P, Saeij JPJ, Carruthers VB, Niles JC, Lourido S. A Genome-wide CRISPR Screen in Toxoplasma Identifies Essential Apicomplexan Genes. Cell. 8;166 (6):1423-1435.e12. doi: 10.1016/j.cell.2016.08.019.<br>Huynh MH, Carruthers VB. Tagging of endogenous genes in a Toxoplasma gondii strain lacking Ku80. Eukaryot Cell. 2009 Apr;8(4):530-9. doi: 10.1128/EC.00358-08.<br><br>293T cell line, ATCC CRL-3216: Brukman, N. G. et al. A novel function for the sperm adhesion protein IZUMO1 in cell-cell fusion. J Cell Biol 222, doi:10.1083/jcb.202207147 (2023).<br>DuBridge, R. B. et al. Analysis of mutation in human cells by using an Epstein-Barr virus shuttle system. Mol Cell Biol 7, 379-387, doi:10.1128/mcb.7.1.379-387.1987 (1987).<br>Pear, W. S., Nolan, G. P., Scott, M. L. & Baltimore, D. Production of high-titer helper-free retroviruses by transient transfection. Proc Natl Acad Sci U S A 90, 8392-8396, doi:10.1073/pnas.90.18.8392 (1993).
Authentication	HFF-1 and 293T cell lines were authenticated by the ATTC using intraspecies STR analysis. Toxoplasma strains were genotyped by PCR and sequenced, as appropriate.
Mycoplasma contamination	HFF human cells and parasites were frequently tested and found negative for Mycoplasma.
Commonly misidentified lines (See ICLAC register)	No commonly misidentified lines were used in this study.

Animals and other research organisms

Policy information about studies involving animals; ARRIVE guidelines recommended for reporting animal research, and Sex and Gender in Research

Laboratory animals	Six-week old BALB/C mice; New-zealand white Rabbit.
Wild animals	none
Reporting on sex	none
Field-collected samples	none
Ethics oversight	The mouse and rabbit experiments were conducted according to the guidelines and regulations issued by the Veterinary Office of China Agricultural University (Issue No. AW11402202-2-1)(Issue No. AW11402202-2-1).

Note that full information on the approval of the study protocol must also be provided in the manuscript.
